# Supplementary material for: Comparative Genomics Analysis Combined with Homologous Overexpression Reveals the Mechanism of Species-Specific Acid Stress Resistance in Bifidobacterium animalis
Source: Foods. 2025 Dec 10;14(24):4243. doi: 10.3390/foods14244243 (PMC12731931; doi:10.3390/foods14244243)
Supplement: Supplementary file 1 [file foods-14-04243-s001.zip › foods-3996188-Table S1.pdf]

Species	Strains	Accession number
Bifidobacterium animalis	*B. animalis* subsp. *lactis* A6	ASM81704v1
	B. animalis subsp. *lactis* RH	ASM69589v1
	B. animalis subsp. *lactis* BF052	ASM81805v1
	B. animalis subsp. *lactis* AD011	ASM2142v1
	B. animalis subsp. *lactis* ATCC 27673	ASM47194v1
	B. animalis subsp. *lactis* B420	ASM27732v1
	B. animalis subsp. *lactis* BB-12	ASM2524v1
	B. animalis subsp. *lactis* Bi-07	ASM27734v1
	B. animalis subsp. *lactis* Bl-04	ASM2270v1
	B. animalis subsp. *lactis* Bl12	ASM41421v1
	B. animalis subsp. *lactis* BLC1	ASM22496v2
	B. animalis subsp. *lactis* CNCM I-2494	ASM22088v1
	B. animalis subsp. *lactis* DSM 10140	ASM2296v1
	B. animalis subsp. *lactis* KLDS2.0603	ASM81620v1
	B. animalis subsp. *lactis* V9	ASM9276v1
Bifidobacterium longum	*B. longum* BXY01	ASM73020v1
	B. longum 105-A	ASM82929v1
	B. longum BG7	ASM129314v1
	B. longum DJO10A	ASM894v1
	B. longum E18	BLONGv1.0
	B. longum NCC2705	ASM752v1
	B. longum BT1	ASM128130v1
	B. longum 157F	ASM19657v1
	B. longum ATCC 15697	ASM2042v1
	B. longum NCIMB8809	ASM144625v1
	B. longum CCUG30698	ASM144627v1
	B. longum BBMN68	ASM16631v1
	B. longum F8	ASM21075v1
	B. longum GT15	ASM77248v1
	B. longum JCM 1217	ASM19655v1
	B. longum JDM301	ASM9232v1
	B. longum KACC 91563	ASM21945v1
Bifidobacterium bifidum	*B. bifidum* BF3	ASM128134v1
	B. bifidum ATCC 29521	ASM102513v1
	B. bifidum BGN4	ASM26509v1
	B. bifidum PRL2010	ASM16590v1
	B. bifidum S17	ASM16496v1
Bifidobacterium breve	*B. breve* BR3	ASM128142v1

B. breve 12L	ASM56895v1
B. breve 689b	ASM56905v1
B. breve ACS-071-V-Sch8b	ASM21386v1
B. breve DSM 20213	ASM102517v1
B. breve JCM 7017	ASM56897v1
B. breve JCM 7019	ASM56901v1
B. breve NCFB 2258	ASM56903v1
B. breve S27	ASM56907v1
B. breve UCC2003	ASM22013v1
