# Supplementary material for: Comparative Genomics Analysis Combined with Homologous Overexpression Reveals the Mechanism of Species-Specific Acid Stress Resistance in Bifidobacterium animalis
Source: Foods. 2025 Dec 10;14(24):4243. doi: 10.3390/foods14244243 (PMC12731931; doi:10.3390/foods14244243)
Supplement: Supplementary file 1 [file foods-14-04243-s001.zip › foods-3996188-Table S2.pdf]

Gene name	Primer name	Primer sequence （5′-3′）
16s rRNA	16sF	CGCCATTGGTGTTCTTCC
	16sR	CCTATTGCGAGCGTGGGA
BAA6_RS02390	2390F	CTTGTCGAGTTTGCCCTTGC
	2390R	GAACGCGACATCGAGGATCT
BAA6_RS02980	2980F	CAAACTCACCCGCAAACGAG
	2980R	GGAACTGCTTCGACCCTTCA
BAA6_RS03885	3885F	TCGTCCTCCTTGGCAATCAC
	3885R	GATCGCATGGAGGAGAAGCA
BAA6_RS05205	5205F	ACAACATGAACAACGGCACG
	5205R	AACCTCGTTCGATTTCGGCT
BAA6_RS06440	6440F	ATACGGGTCTTCCCACTGGT
	6440R	CTGACCATCCGTACACCGAC
BAA6_RS06240	6240F	GACCGCGAATCCAAATGGTG
	6240R	GAGGAACATCGGCACGAGAA
BAA6_RS00480	480F	GATGAAGCTGACGATGGCGA
	480R	GTGCGATTACCAACAACGGG
BAA6_RS06435	6435F	GTCTGGAAATAGAGCGGCGA
	6435R	TGATCCGCGACGATTACCTG
BAA6_RS02185	2185F	AACATCTGCGCACACTACGA
	2185R	TCAGTCGAACAACGTCCAGG
BAA6_RS06445	6445F	CTCGGCGACTTCCTTCTTGT
	6445R	GAAGAGATCAAGGCCCGCAT
