# Supplementary material for: Comparative Genomics Analysis Combined with Homologous Overexpression Reveals the Mechanism of Species-Specific Acid Stress Resistance in Bifidobacterium animalis
Source: Foods. 2025 Dec 10;14(24):4243. doi: 10.3390/foods14244243 (PMC12731931; doi:10.3390/foods14244243)
Supplement: Supplementary file 1 [file foods-14-04243-s001.zip › foods-3996188-Table S3.pdf]

Gene name	Primer name	Primer sequence （5′-3′）	Restriction Enzyme cutting site
BAA6_RS02390	2390exF	TCCCCCGGGATGGCAGAAGACAAGACC	*Sma*I
	2390exR	CCCAAGCTTCTACTTTGCGAGGGAAT	*Hin*dIII
BAA6_RS02980	2980exF	TCCCCCGGGATGAAGGTGCTTTGCGCT	*Sma*I
	2980exR	CCCAAGCTTTTACTCCCCCCG	*Hin*dIII
BAA6_RS03885	3885exF	TCCCCCGGGATGGCAGACATCAAGGAAC	*Sma*I
	3885exR	CCCAAGCTTTCAGCCGAGCTGGGTTCC	*Hin*dIII
BAA6_RS05205	5205exF	TCCCCCGGGATGGAATGGGCAGCA	*Sma*I
	5205exR	CCCAAGCTTTCACTCGGCCTGCCCGCC	*Hin*dIII
BAA6_RS06440	6440exF	TCCCCCGGGATGGCAGAGCAACAGAACA	*Sma*I
	6440exR	CCCAAGCTTTCACTTGTCTTCGAGAT	*Hin*dIII
BAA6_RS06240	6240exF	TCCCCCGGGATGACCAGTATTTTCG	*Sma*I
	6240exR	CCCAAGCTTCTAGAGCAGGCAGGCAACC	*Hin*dIII
BAA6_RS00480	480exF	TCCCCCGGGATGTTTTGGATTTGGACACT	*Sma*I
	480exR	CCCAAGCTTCTATGCGCGAACCTTCA	*Hin*dIII
BAA6_RS06435	6435exF	CCAATGCATCGCGTAGTGTCACGCAAAC	*Nsi*I
	6435exR	CCCAAGCTTTCAGTTACTTGCGTCA	*Hin*dIII
BAA6_RS02185	2185exF	CCAATGCATATGGCATTGACCAGCGAA	*Nsi*I
	2185exR	CCCAAGCTTTCAGTCGAACAACGTCCA	*Hin*dIII
BAA6_RS06445	6445exF	TCCCCCGGGATGGTTGATATGAGCGA	*Sma*I
	6445exR	CCCAAGCTTTCAGTGGGTCTGGTGGGT	*Hin*dIII
